# Casimir force phase transitions in the graphene family

**DOI:** 10.1038/ncomms14699

**Published:** 2017-03-15

**Authors:** Pablo Rodriguez-Lopez, Wilton J. M. Kort-Kamp, Diego A. R. Dalvit, Lilia M. Woods

**Affiliations:** 1 Department of Physics, University of South Florida, Tampa Florida, 33620, USA; 2 Center for Nonlinear Studies, MS B258, Los Alamos National Laboratory, Los Alamos, New Mexico 87545, USA; 3 Theoretical Division, MS B213, Los Alamos National Laboratory, Los Alamos, New Mexico 87545, USA

## Abstract

The Casimir force is a universal interaction induced by electromagnetic quantum fluctuations between any types of objects. The expansion of the graphene family by adding silicene, germanene and stanene (2D allotropes of Si, Ge, and Sn), lends itself as a platform to probe Dirac-like physics in honeycomb staggered systems in such a ubiquitous interaction. We discover Casimir force phase transitions between these staggered 2D materials induced by the complex interplay between Dirac physics, spin-orbit coupling and externally applied fields. In particular, we find that the interaction energy experiences different power law distance decays, magnitudes and dependences on characteristic physical constants. Furthermore, due to the topological properties of these materials, repulsive and quantized Casimir interactions become possible.

## Abstract (web summary)

The Casimir force is a ubiquitous interaction arising from electromagnetic quantum fluctuations. Here, the authors uncover the underlying physics governing Casimir force phase transitions in staggered 2D materials in the graphene family.

Interactions originating from electromagnetic quantum fluctuations are universal as they exist between objects regardless of their specific properties or boundary conditions. These ubiquitous interactions lead to the well-known van der Waals (vdW) force^1^ when the exchange of electromagnetic fluctuations can be considered instantaneous, and to the Casimir and Casimir–Polder forces when the distances between the objects are large and the finite speed of light is important^2^,^3^. Although these interactions are typically weak, they have appreciable effects at nano- and micro-metre separations. For example, adhesion, stiction, wetting and stability of materials composed of chemically inert constituents occur due to vdW/Casimir interactions^4^,^5^,^6^,^7^. The discovery of systems with reduced dimensions and physics different from the one of standard three-dimensional dielectrics, metals and semiconductors has given a new impetus to the field of vdW/Casimir phenomena. Specifically, systems involving graphene^8^ have a strong dependence on temperature and doping in their vdW/Casimir interactions^9^,^10^,^11^,^12^,^13^,^14^,^15^. Experimental measurements have demonstrated that the vdW force between substrates is almost completely screened when one is covered by graphene^16^, while temperature effects in graphene-based Casimir interactions have also been reported^17^.

Recently, the graphene family has expanded. Silicon, germanium and tin, being in the same column of the periodic table as carbon, also have stable two-dimensional (2D) layers^18^,^19^,^20^. Unlike the planar sp^2^ bonded graphene, silicene, germanene and stanene have spatial buckling between the two sublattices caused by their stronger sp^3^ bonding. These newer members of the 2D graphene family exhibit non-trivial topological insulator features. The application of external fields together with the inherently strong spin-orbit coupling can be used as effective ‘knobs' for various Hall transitions^21^,^22^,^23^,^24^,^25^,^26^,^27^,^28^,^29^,^30^. Furthermore, vertically stacking of different 2D materials held by vdW interactions is emerging as a new scientific direction, where desired properties by design can be achieved^31^,^32^. Recent studies have shown that the vdW interactions affect the electronic and phonon properties of such vdW heterostructures^33^,^34^, which is especially relevant for their transport and optical applications.

In this paper, we study the physics of Casimir interactions in the graphene family, which serve as a platform for probing low-energy Dirac-like physics in systems that can experience different Hall transitions. We find that phase transitions between the various electronic phases in these materials, attained by means of externally applied circularly polarized lasers and/or static electric fields, strongly impact fluctuation-induced phenomena. Novel distance scaling laws, abrupt magnitude changes, force quantization and repulsion are all manifestations of Casimir force phase transitions occurring in these 2D staggered materials.

## Results

### Electro-optical response of the 2D graphene family

**Figure 1:**
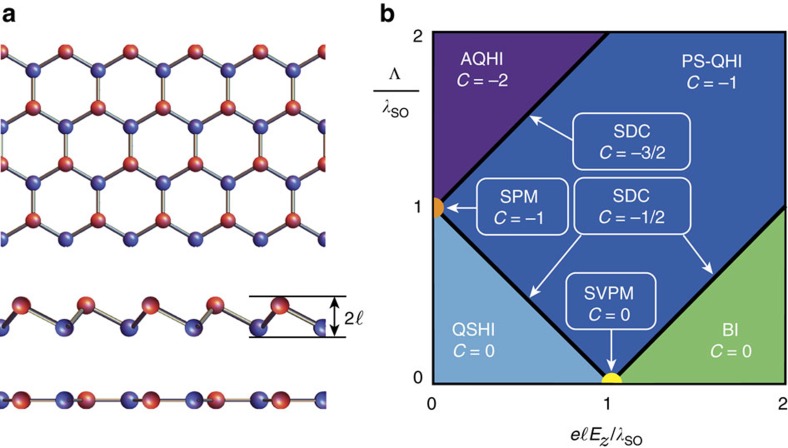
Phase diagram of the graphene family. (**a**) Top view of the hexagonal lattice structure of the graphene family. The red and blue colours represent the two inequivalent atoms in the structure. While graphene has planar atomic configuration (side view shown), the graphene family materials (silicene, germanene and stanene) have a finite staggering 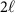 between the two sublattices. (**b**) First quadrant of the phase diagram of the graphene family materials in the 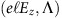 plane in units of *λ*_SO_^24^. The distinct electronic phases (acronyms are defined in the main text) are characterized by the Chern number *C*.

Silicene, germanene and stanene have layered honeycomb structure similar to graphene, but the two inequivalent atoms in the unit cell are arranged in staggered layers characterized by a finite buckling 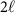, as shown in Fig. 1a^21^,^25^,^35^. In graphene, artificial efforts are needed to modify the carrier mass and induce spin-orbit coupling (SOC)^36^,^37^. However, thanks to the buckling and heavier constituent atoms, such properties are already intrinsic to silicene, germanene and stanene. The low energy band structure can be determined from a Dirac-like Hamiltonian, obtained from a nearest neighbour tight binding model, which also includes an external electric field *E*_*z*_ perpendicular to the 2D plane of the material and irradiated circularly polarized light^24^,^38^


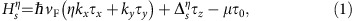



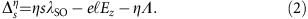


Here *τ*_*i*_ are the Pauli matrices for the sublattice pseudospin index *η*=±1, *τ*_0_ is the identity matrix and the spin index *s*=±1 denotes the eigenvalues of the Pauli spin matrix *σ*_*z*_. Also, *e* is the electron charge, *μ* is the chemical potential, 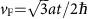 is the Fermi velocity, where *a* is the lattice constant (*a*^Gra^=2.46 Å, *a*^Sil^=3.86 Å, *a*^Ger^=4.02 Å and *a*^Stan^=4.7 Å) and *t* is the nearest-neighbour coupling (*t*^Gra^=2.8 eV, *t*^Sil^=1.6 eV, *t*^Ger^=1.3 eV and *t*^Stan^=1.3 eV). For graphene, 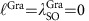, and for the other materials, 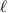 has values that are of similar order (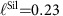 Å, 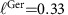 Å and 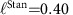 Å), but *λ*_SO_ can vary by orders of magnitude (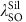
=3.9 meV, 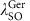
=43 meV and 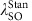
=100 meV)^38^. The components of the 2D wave vector in equation (1) are denoted as *k*_*x*,*y*_ and 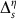 is the Dirac mass at the *K*_*η*_ points for each spin index *s*, characterized by the eigenenergy 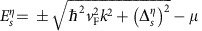.

The mass parameter in equation (2) depends on the strength of the SOC and the spin and valley degrees of freedom of the carriers. It can be further controlled by *E*_*z*_, which generates an electrostatic potential 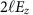 between the two different atoms in the unit cell. Other types of SOC originating from Rashba physics, such as the Rashba SOC associated with the next-nearest neighbour hopping and the Rashba SOC associated with the nearest neighbour hopping induced by *E*_*z*_, are neglected here due to their small effects as compared to *λ*_SO_ (ref. 23). The properties of all 2D materials can also be modified by irradiating circularly polarized light, with the electromagnetic vector potential given by **A**(*t*)=*A*_0_(cos(*ω*_0_*t*), ±sin(*ω*_0_*t*), 0), where *A*_0_ is an amplitude and *ω*_0_ is the frequency of the applied light with +(−) specifying left (right) circular polarization. In the limit 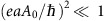, and using a low-energy Hamiltonian approach, this results in a contribution to the Dirac mass gap given by Λ=±(*ev*_F_*A*_0_)^2^/*c*^2^*ħω*_0_ (we use cgs electromagnetic units)^24^, as shown in equation (2). We should note that the light field may also cause additional coupling between the energy bands^39^, which can open gaps in the band structure typically at energies around *nħω*_0_/2 (*n*=±1, ±2, etc). Hence, the Hamiltonian in equation (1) is valid as long as 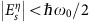.

The staggered 2D layers exhibit several electronic phases^22^,^24^ resulting from changes in 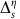 induced by *E*_*z*_ and/or Λ (see Fig. 1b). At *E*_*z*_=Λ=0, the 2D layer can be characterized as a quantum spin Hall insulator (QSHI). Fixing Λ=0 and increasing *E*_*z*_, it remains in the QSHI phase until the critical electric field 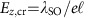 is reached. At this point, two Dirac cones are closed 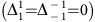 and the material becomes a spin valley polarized semimetal (SVPM). Further increasing the electric field *E*_*z*_>*E*_*z*,cr_, the magnitude of all four 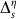 increases and the 2D layer becomes a regular band insulator (BI). In the case that we fix *E*_*z*_=0 and increase Λ, the system goes through a phase transition from the QSHI phase to a spin polarized metal (SPM) phase at the critical value Λ_cr_=*λ*_SO_, where the energy gap of one of the spins closes. For Λ>Λ_cr_, the anomalous quantum Hall insulator (AQHI) phase is reached. When both *E*_*z*_ and Λ are non-zero, these materials can have other topological phases^24^. For example, the region of the phase diagram in Fig. 1b) where 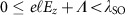 corresponds to a QSHI phase. Along the line 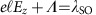, it is possible to have only one Dirac cone closed, the single Dirac cone (SDC) phase. Finally, when the conditions 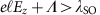 and 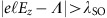 are simultaneously satisfied, the closed gap opens again but with the opposite sign resulting in a polarized spin quantum Hall insulator (PS-QHI) state, a combination of the AQHI and QSHI phases. For completeness, we briefly describe the other three quadrants of the phase diagram in Fig. 1b. The second quadrant (*E*_*z*_<0, Λ>0) is obtained from the first one by taking its mirror replica with respect to the *E*_*z*_=0 axis. The third and fourth quadrants are obtained by taking the mirror replica of the first two with respect to the Λ=0 axis and inverting the signs of the Chern numbers.

**Figure 2:**
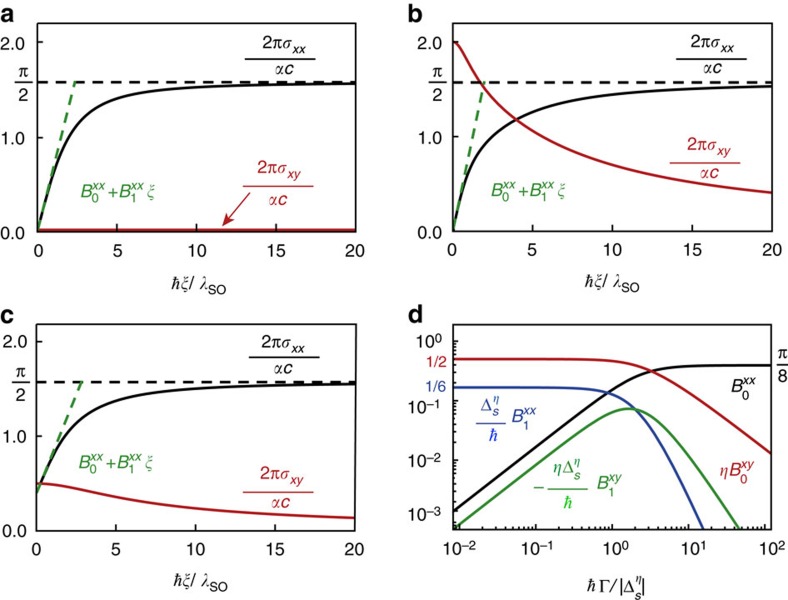
Zero-temperature longitudinal and Hall conductivities at imaginary frequencies. The behaviour of *σ*_*xx*_(*iξ*) and *σ*_*xy*_(*iξ*) for different phases is shown: (**a**) *E*_*z*_=Λ=0 (QSHI phase with *C*=0); (**b**) Λ/*λ*_SO_=−3/2 and *E*_*z*_=0 (AQHI phase with *C*=2); and (**c**) 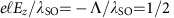 (SDC phase with *C*=1/2). In all cases, *μ*=Γ=0. The horizontal black dashed line is *σ*_*xx*_(*iξ*→∞)=*αc*/4. The dashed green lines correspond to the low-frequency expansion for the conductivities given in equation (4). For *μ*=0, 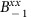 vanishes identically, while the other coefficients 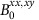 and 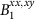 are shown in **d** as a function of *ħ*Γ/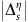.

The energy band structure has important consequences for the electro-optical response, and in particular for the conductivity tensor at imaginary frequencies, needed for the Casimir force computation (see below). Using the standard Kubo formalism^40^,^41^, we obtain the zero-temperature dynamical 2D conductivity tensor *σ*_*ij*_(*iξ*, 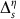) of each Dirac cone. Here, *iξ* is an imaginary frequency, and *i*, *j*=*x*, *y* are Cartesian components. For the inter-plate separations and temperatures we study below, effects of spatial dispersion can be neglected^10^,^42^. The dynamical conductivity components due to intraband 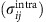 and interband 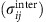 transitions are found to be


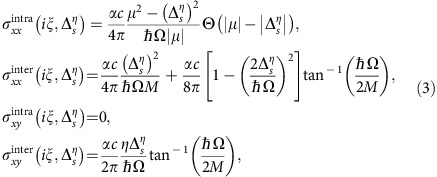


where *σ*_*yy*_(*iξ*, 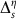)=*σ*_*xx*_(*iξ*, 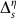) and *σ*_*yx*_(*iξ*, 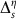)=−*σ*_*xy*_(*iξ*, 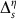). Here *α*=*e*^2^/*ħc*≈1/137 is the fine structure constant, 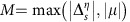 and Ω=*ξ*+Γ, where Γ=1/2*τ* with *τ* being the relaxation scattering time. Corresponding expressions for the silicene optical conductivity at real frequencies have already been reported in the literature^29^,^30^. The dynamical conductivity from all Dirac cones, necessary for the evaluation of the Casimir interaction, is 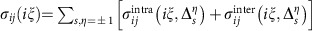. The various topological phases associated with the Hall effect, displayed in Fig. 1b, are captured via the *η*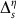 product in *σ*_*xy*_. In Fig. 2a–c, we show the different elements of the conductivity tensor as a function of imaginary frequency at various points in the phase diagram. Finite temperature effects on the optical conductivity are outlined in Supplementary Note 2 and given in Supplementary Figs 1 and 2.

### Low frequency optical response

Since the Casimir interaction at large separations is determined mainly by the low frequency response^7^, understanding the optical conductivity at *iξ*=0 is particularly important. We first consider the case Λ=0. Graphene has neither staggering nor SOC, and hence 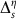
=0 for all cones. Using equation (3), one recovers the well-known result^43^ for the graphene universal conductivity *σ*_*xx*_(*iξ*)=*αc*/4 and *σ*_*xy*_(*iξ*)=0 in the non-dissipative limit. For the other members of the graphene family, their conductivity tensors can be cast into the perspective of a Chern insulator description (see Fig. 1b), in which the corresponding Chern number is given by 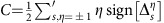, and captures the topologically non-trivial features of these 2D materials^23^,^24^. The prime in the summation indicates that only terms with 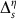
≠0 should be included. Let us now consider the case for 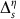
≠0 and, as above, restrict ourselves to the dissipationless limit (Γ=0). When 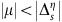, we find that *σ*_*xx*_(*iξ*=0, 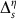)=0 and 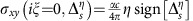 for each cone. Thus the total Hall conductivity is 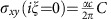, which explicitly connects with the Chern insulator topological nature of these materials via the particular electronic phase. For example, the *C*=0 for the QSHI phase at *E*_*z*_=0 results in *σ*_*xy*_(*iξ*=0)=0 (Fig. 2a). The *C*=2 AQHI phase at Λ/*λ*_SO_=−3/2 leads to 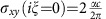 since there are four open Dirac cones and each contributes with the same sign to the Hall conductivity (Fig. 2b). The *C*=1/2 SDC phase at 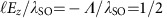 gives *σ*_*xy*_(*iξ*=0)=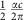 since there are three open Dirac cones (Fig. 2c).

To gain further insight into the various factors affecting the contribution of each single Dirac cone to the conductivity *σ*_*ij*_(*iξ*, 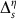), we perform a low-frequency expansion. Using equation (3), one finds


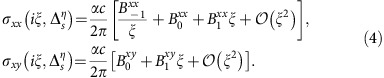


The coefficients 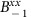, 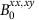, and 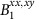 are a function of the parameters of the 2D material (that is, 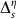, Γ and *μ*), and their explicit expressions are given in Supplementary Note 1. It is interesting to note that each term in equation (4) is reminiscent of a particular dielectric response function. For example, the first term of the longitudinal conductivity behaves as the plasma model for metals with 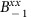 specifying the plasma frequency, and it originates entirely from intraband transitions. The Lorentz model for dielectrics is recognized in the third term with 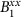 giving the strength of the Lorentz oscillator. 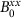
corresponds to a constant conductivity. On the other hand, 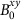 captures the Hall effects in the 2D materials, and in the lossless case, it can be written as 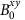
=*C*, which shows the quantized nature of the Hall conductivity via the Chern number. Figure 2a–c shows how the above low-frequency expansion for the longitudinal conductivity compares to the full Kubo expression.

For the case *μ*=0, 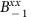 identically vanishes, and the remaining coefficients are shown in Fig. 2d. When 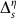
=0, 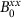
=*π*/8 and 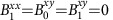 for all values of the dissipation parameter. When 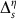
≠0, dissipation influences the coefficients. In the limit of small dissipation, 
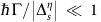
, 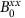
≈*ħ*Γ/6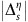, 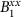
≈*ħ*/6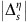, 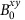
≈*η* sign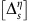/2 and 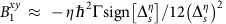. In the opposite limit, 
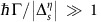
, 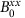
=*π*/8 and all other coefficients tend to zero.

### Casimir force phase transitions

The Casimir energy per unit area 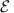 and the corresponding Casimir force 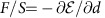 between two layers of area *S* of the graphene family materials separated by a distance *d* can be calculated using the continuum Lifshitz approach, which applies for separations larger than several times the interatomic distances in the involved objects^7^. We first discuss the effects of quantum (zero-temperature) fluctuations on the Casimir energy, which in this case is expressed as an integral over complex frequencies *iξ* (see Methods section). Beginning with neutral (*μ*=0) graphene/graphene interaction, the Casimir energy per unit area is found to be


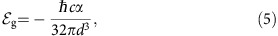


and results in Casimir attraction^9^,^10^,^11^. Compared with the Casimir energy for perfect metals 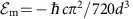, it reveals that although the distance dependence is the same, the magnitude of 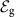 is much reduced due to the presence of *α*.

**Figure 3:**
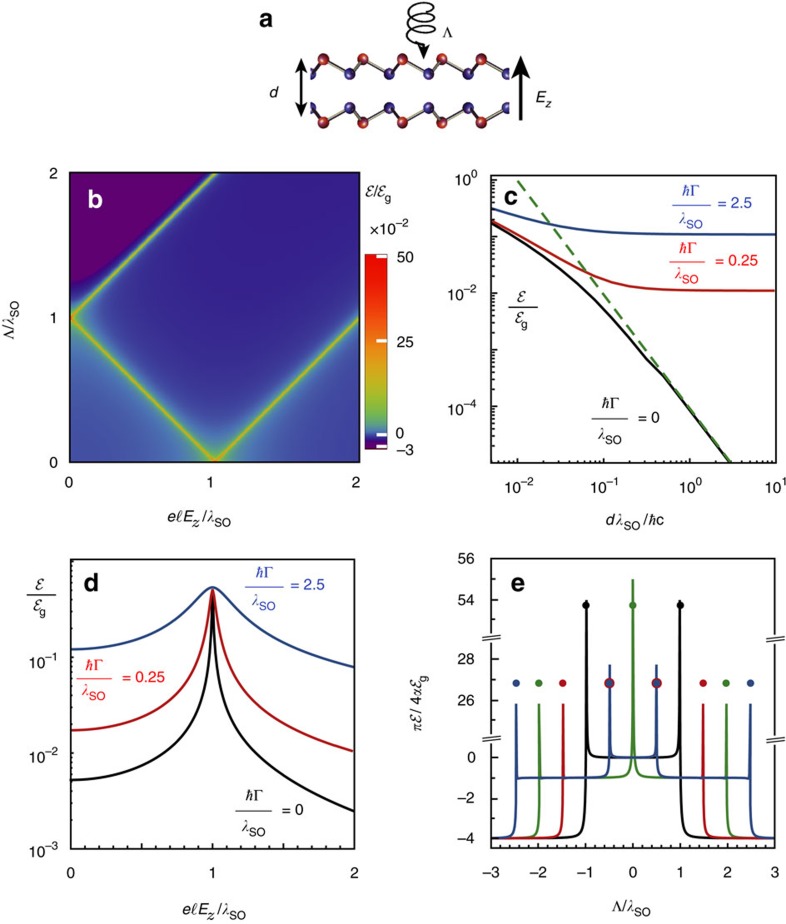
Zero-temperature Casimir interaction in the graphene family. (**a**) Fabry–Pérot cavity formed by two layers of the graphene family materials under externally applied fields. (**b**) Casimir energy phase diagram for two dissipationless identical parallel layers for *dλ*_SO_/*ħc*=1. (**c**) Distance dependency of the Casimir energy at Λ=*E*_*z*_=0 for various values of dissipation. (**d**) Effect of the external electric field on the Casimir energy at Λ=0 for different values of dissipation at a distance *dλ*_SO_/*ħc*=1. (**e**) Quantized and repulsive Casimir energy for two dissipationless identical layers at *d*=10*ħc*/*λ*_SO_. The various curves correspond to different values of the electric field, 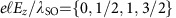 (black, red, green and blue, respectively). In the large-distance asymptotics (Table 1), the rounded plateaus become abrupt jumps and the interaction energies at phase transition boundaries are the dots in-between plateaus. In all plots, *μ*=0.

Probing the expanded graphene family optical response by changing *E*_*z*_ and/or Λ results in a much richer Casimir interaction picture. The competition between *σ*_*xx*_ and *σ*_*xy*_ dominance and the relative contribution of the different coefficients 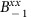, 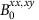 and 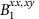 result in many different asymptotic scaling laws, significant magnitude changes, force quantization and repulsion. We consider a Fabry–Pérot cavity formed by two sheets of the graphene family (for example, Sil/Sil, Ger/Ger and Stan/Stan) (Fig. 3a). As for graphene, each staggered layer is almost transparent to the incident light (transmission coefficient *T*≃1−*πα*), and hence both layers forming the cavity experience irradiation essentially with the same characteristics captured by Λ.

The impact of the different phases of the graphene family materials on the Casimir interaction is shown in Fig. 3b for *μ*=Γ=0 and a distance *d*=*ħc*/*λ*_SO_. The Casimir energy density plot reflects the phase diagram of Fig. 1b. Note that, for the parameters of the figure, 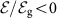 in most of the AQHI and PS-QHI phases and will ultimately result in Casimir force repulsion (see discussion below about Fig. 3e). On the other hand, in all other phases 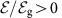 corresponding to attraction. As one approaches phase transition boundaries, the Casimir energy significantly increases in magnitude featuring a cusp-like behaviour (see black curve in Fig. 3d). At shorter distances (where non-zero imaginary frequencies become relevant), the energy phase diagram is modified with less defined phase boundaries (not shown). The dependence of the Casimir interaction energy on separation is shown in Fig. 3c at the origin of phase space Λ=*E*_*z*_=0. The asymptotic result from Table 1 for Γ=0 is given by the green dashed line in the figure. Increasing dissipation in the materials results in a blurring of the phase boundaries and 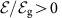 for all phases at large separations (see Supplementary Note 3 and the corresponding Supplementary Fig. 4). The behaviour of the phase diagram along the Λ=0 line for different values of dissipation is shown in Fig. 3d. The Casimir energy for lossless QSHI–QSHI or BI–BI phase combinations changes to that corresponding to the SVPM–SVPM configuration as *E*_*z*_ approaches *E*_*z*,cr_, presenting a cusp-like feature. At either side of the cusp, all Dirac masses are non-zero, while right at the cusp two Dirac cones close. The interaction energy in the SVPM–SVPM configuration has a graphene-like behaviour (see Table 1) but, since two rather than four gaps are closed, there is a 50% magnitude reduction, namely, 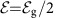. When losses are included, the cusp-like feature is rounded and the interaction increases.

Analytical expressions for the large distance asymptotics 
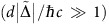
 of the zero temperature Casimir interaction are summarized in Table 1 for a given combination of phases in the interacting materials (assumed to have *μ*=0 and the same Γ), both for the case of zero and small dissipation. Each of the entries in the table can be obtained by the following procedure. First, one determines whether a mass gap closes for either of the phases, and then one identifies the corresponding relevant coefficients 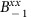, 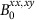 and 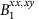. Given this information, one computes the large-distance Casimir energy 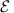 (see Methods section) to leading order in the fine structure constant and the distance-decay power, using for the product of the reflection matrices **R**_1_·**R**_2_ the appropriate combinations of *B* coefficients for each of the interacting materials. Let us first discuss the case of zero dissipation. When the staggered layers are either in the QSHI or BI phase, all mass gaps are non-zero, the relevant coefficient is 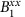 (note that for these phases 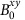 vanishes upon summing over valley and spin indices) and the Casimir energy scales as 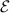
∼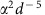. This dependency upon *α*^2^ suggests a much weaker interaction as compared to two graphene sheets (see equation (5)). When one of the materials is either in the QSHI or BI phase, while the other one is in the SPM or SVPM phase, two mass gaps are closed, the relevant coefficients are 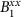 (for the material in the QSHI/BI phase) and 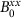
=*π*/8 (for the material in the SPM/SVPM phase), and the energy scales as 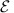
∼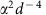. However, when the SPM or SVPM phase is substituted by an AQHI or PS-QHI phase, the Hall coefficient 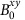 becomes relevant, and the asymptotic Casimir energy is found to be 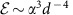. Finite-dissipation corrections 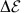 to the large-distance Casimir energy are governed by the coefficient 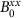 in all phases. Analytical expressions for this correction can be obtained in the limit of small dissipation, 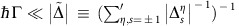. As shown in Table 1, 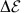 inherits the linear in Γ dependency from 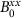 (see Fig. 2d) and decays as *d*^−3^ for all phase combinations. As compared to the lossless case, dissipation results in a qualitative change of the power-law decay of the interaction, in sharp contrast to the situation of typical three-dimensional planar slabs where dissipation only scales the large-distance Casimir energy by an overall numerical factor.

A further striking consequence of the different electronic phases in the graphene family is that the Casimir energy can be quantized. Since the large-distance zero temperature interaction energy between lossless 2D staggered layers in AQHI, PS-QHI or SPM phases is proportional to their Hall conductivities and 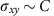, we find that 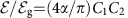, that is, the Casimir energy is quantized in terms of the product of Chern numbers (see Table 1). At this point, it is important to emphasize that the reflection matrices entering the Lifshitz formula correspond to reflection of vacuum fluctuations from within the Fabry–Pérot cavity and that the sign of the Hall conductivities on either layer (induced by the external circularly polarized laser) changes as seen from fluctuations impinging on the bottom or top layer. The overall result is that the signs of the Chern numbers of the bottom and top layers are different, *C*_1_*C*_2_<0, and hence the Casimir force is not only quantized but is also repulsive. This is shown for the case of two dissipationless AQHI or PS-QHI identical sheets in Fig. 3e. The zero-temperature Casimir energy features a ladder-like quantized and repulsive behaviour of the Casimir energy 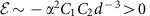 with the strongest repulsion for *C*_1_=−*C*_2_=±2. A physical picture of this large-distance Casimir repulsion can be obtained by noting that the polarized laser field induces circulating currents on both layers, whose sense of rotation is determined by the sign of the Hall conductivities. The Casimir cavity is essentially a collection of current loops on each layer facing each other or, equivalently, two parallel sheets of magnetic dipoles. Recalling that anti-parallel magnetic dipoles repel, it follows that two AQHI, PS-QHI or SPM layers with Hall conductivities of unequal sign will repel. For the other SDC/SVPM/SPM phases, on the other hand, the Casimir energy behaves as 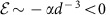, which corresponds to the attractive force between two semimetals, and in the large-distance asymptotics results in an abrupt change of the Casimir force. The QSHI/BI phases also result in an attractive force but with a stronger decay, 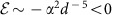. Results for other combination of materials of the graphene family (for example, silicene–graphene and silicene–germanene) as well as effects of finite dissipation in the quantized repulsive Casimir force are shown in Supplementary Note 3 and depicted in Supplementary Fig. 5.

We briefly discuss the effect of the chemical potential. As long as 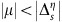 for all Dirac cones, the results described above for the *μ*=0 case still hold. When 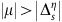 for at least one Dirac cone, the intraband conductivity (equation (3)) starts to play a role. For Γ=0, the low-frequency optical response is dominated by the plasma-like term in equation (4) containing 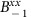, and the large-distance Casimir energy corresponds to that of a perfect conductor. For Γ>0, 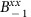
=0 and the dominant contribution to the large-distance Casimir energy comes from 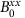. In the limit 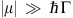, the Casimir energy corresponds to that of 2D Drude metals. Further details of the effect of *μ* on Casimir force phase transitions can be found in Supplementary Note 3 and Supplementary Fig. 3.

### Thermal corrections to the Casimir energy

**Figure 4:**
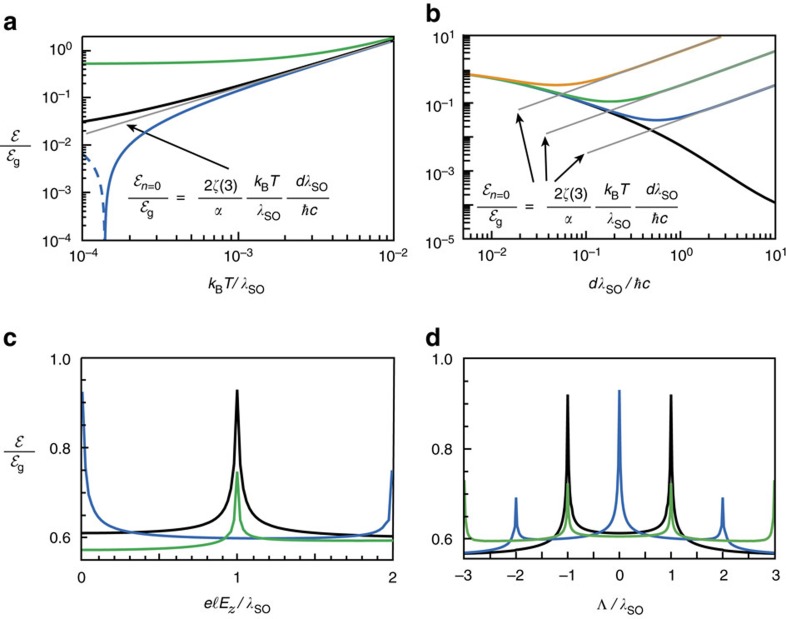
Finite-temperature Casimir interaction in the graphene family. (**a**) Casimir energy between identical layers of the graphene family as a function of temperature for some representative points 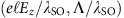 in phase space: (0, 0) (black), (0, 1) (green), and (0,2) (blue; dashed blue corresponds to repulsion, where 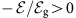). The distance is fixed at *dλ*_SO_/*ħc*=0.5. (**b**) Distance dependency of the Casimir energy at the origin of phase space *E*_*z*_=Λ=0 for various temperatures *k*_B_*T*/*λ*_SO_=0 (black), 10^−4^ (blue), 10^−3^ (green) and 10^−2^ (orange). The thin grey curves in panels (**a**,**b**) denote the *n*=0 Matsubara contribution to the energy (see Supplementary Information). (**c**) Cut of the Casimir energy phase diagram along Λ/*λ*_SO_=0 (black), 1 (blue) and 2 (green) as a function of the electric field. (**d**) Cut along 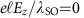 (black), 1 (blue) and 2 (green) as a function of Λ/*λ*_SO_. In both panels (**c**,**d**), the temperature is *k*_B_*T*/*λ*_SO_=3.6 × 10^−3^ (corresponding to 4.2 K for stanene), and the distance is fixed at *dλ*_SO_/*ħc*=0.5 (corresponding to 940 nm for stanene). In all plots, *μ*=0, *ħ*Γ/*λ*_SO_=10^−4^ and the normalization 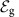 is the zero-temperature graphene energy given in equation (5).

Thermal effects enter in the Lifshitz formula by replacing the integral over complex frequencies with a summation over Matsubara frequencies and taking into account the finite-temperature conductivity (see Methods section, Supplementary Note 4 and Supplementary Figs 1 and 2). In Fig. 4a we show the Casimir energy between identical layers of the graphene family as a function of temperature at a fixed distance for some representative points in phase space. For low temperatures 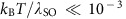, Casimir repulsion for the AQHI–AQHI phases is still present (dashed blue curve), and as the temperature increases there is a cross-over to attraction. Another effect of temperature is to reduce the contrast in magnitude between Casimir energies for different points in phase space (for example, SPM and QSHI phases shown in green and black, respectively), which ultimately results in blurred Casimir force phase transitions. For large temperatures 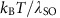
≳10^−2^, all curves are essentially described by the classical limit 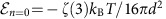, which is the same for all points in phase space (see Supplementary Note 4). Figure 4b depicts the distance dependence of the Casimir energy for the QSHI phase for various temperatures, showing a change of scaling law from 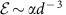 for 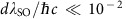 to 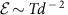 for 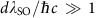. Figure 4c,d show different cuts of the Casimir energy phase diagram for fixed temperature and distance. Thermal effects result in the smoothing out of phase transition boundaries and disappearance of quantized and repulsive Casimir interactions. For example, for the case of a stanene cavity maintained at liquid helium temperature *T*=4.2 K (corresponding to 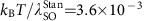), Casimir force phase transitions are still observable in the smoothed cusp-like features.

## Discussion

Our study shows that in order to probe the Dirac-like physics in the graphene family via the rich structure of its Casimir interactions, low temperature set-ups, such as the cryogenic atomic force microscopy developed in ref. 44 to measure Casimir force gradients using a metallic spherical tip, are required. In order to suggest possible experimental signatures of the Casimir force phase transitions, let us consider a stanene layer (neutral and weakly dissipative 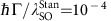) under varying static field along the Λ=0 line in Fig. 1b in front of bulk gold semi-infinite substrate. Evaluating the Casimir pressure at liquid helium temperature *T*=4.2 K and at a distance of *d*=100 nm, we obtain *P*_QSHI-Au_≃0.2 Pa at *E*_*z*_=0 and *P*_SVPM-Au_≃0.3 Pa at 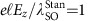. In the proximity force approximation (valid for 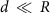, where *R* is the radius of curvature of the metallic sphere), the respective Casimir force gradients *F*′/*R*≈2*πP* are 1.3 and 1.9 Pa. Given the reported sensitivities for *F*′/*R* of 0.1 Pa^44^, it should be possible to probe Casimir force phase transitions in this set-up.

It is worth noting that, when there is an applied polarized laser (Λ≠0), there is an additional optical force on top of the Casimir interaction between the two parallel layers of the graphene family. A straightforward calculation of the optical pressure to leading order in *α* gives 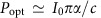, where *I*_0_ is the laser intensity. Typical laser parameters for which the low-energy Hamiltonian equation (1) is valid and for which the phase diagram in Fig. 1b can be explored result in an optical force larger than the Casimir one. Nevertheless, it is still possible to distinguish between the two forces by taking advantage of the particular dependency of the optical force on the laser parameters. For example, modulating the laser polarization between circular (Λ≠0) and linear (Λ=0, since linearly polarized light does not break time reversal symmetry^45^) states and employing a lock-in technique at the modulation frequency, the optical force is removed from the signal (as it is independent of the state of polarization), and one can detect the difference between the Casimir force at (*E*_*z*_, Λ) and at (*E*_*z*_, 0). This measurement, in conjunction with an independent detection of the force for no applied laser field, allows the determination of the Casimir force as a function of distance at any point (*E*_*z*_, Λ) in the phase diagram, irrespective of the strength of the optical force.

We have shown that the Casimir interaction in materials of the graphene family has a rich structure due to their unique electronic and optical properties. Their various electronic phases, tunable by external fields, result in Casimir force phase transitions featuring different distance scaling laws, significant magnitude changes, force quantization and repulsion. The measurement of some of these effects should be within reach with current state-of-the-art low-temperature Casimir force experiments.

## Methods

### Calculation of the interaction energy

The Casimir interaction energy per unit area between two parallel plates separated by a distance *d* at temperature *T* can be calculated using the Lifshitz formula^4^,^7^


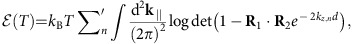


where the summation is over Matsubara frequencies *ξ*_*n*_=2*πnk*_B_*T*/*ħ* (*n*=0, 1, 2, …) and the prime indicates that the *n*=0 term has a 1/2 weight. Furthermore, 
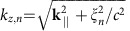
 and 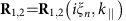 are 2 × 2 reflection matrices. The *T*=0 formula is obtained by the replacement 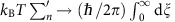. The diagonal elements of the reflection matrices are the *R*_ss_ and *R*_pp_ Fresnel coefficients, and the off-diagonal elements *R*_sp,ps_ arise from the Hall conductivity that induces polarization conversion. Imposing standard boundary conditions to Maxwell's equations for a single 2D sheet, one finds^46^


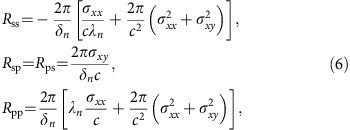


where 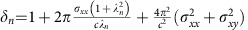, *λ*_*n*_=*k*_*z*,*n*_*c*/*ξ*_*n*_ and the conductivity tensor is evaluated at the imaginary Matsubara frequencies *σ*_*ij*_(*iξ*_*n*_). Note that in the Lifshitz formula the Hall conductivities on either plate must have opposite signs since **R**_*j*_ correspond to reflections within the Fabry–Pérot cavity. In the estimation of the Casimir pressure between stanene and a gold bulk, we model the permittivity of Au as 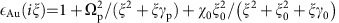, with (Ω_p_, *γ*_p_, *ξ*_0_, *γ*_0_)=(13.7, 0.05, 20, 25) × 10^15^ rad s^−1^ and *χ*_0_=5 (ref. 44).

### Data availability

The data that support these findings are available from the corresponding authors on request.

## Additional information

**How to cite this article:** Rodriguez-Lopez, P. *et al*. Casimir force phase transitions in the graphene family. *Nat. Commun.* **8,** 14699 doi: 10.1038/ncomms14699 (2017).

**Publisher's note**: Springer Nature remains neutral with regard to jurisdictional claims in published maps and institutional affiliations.

## Supplementary Material

Supplementary Information Supplementary Notes and Supplementary Figures

## Figures and Tables

**Table 1:**
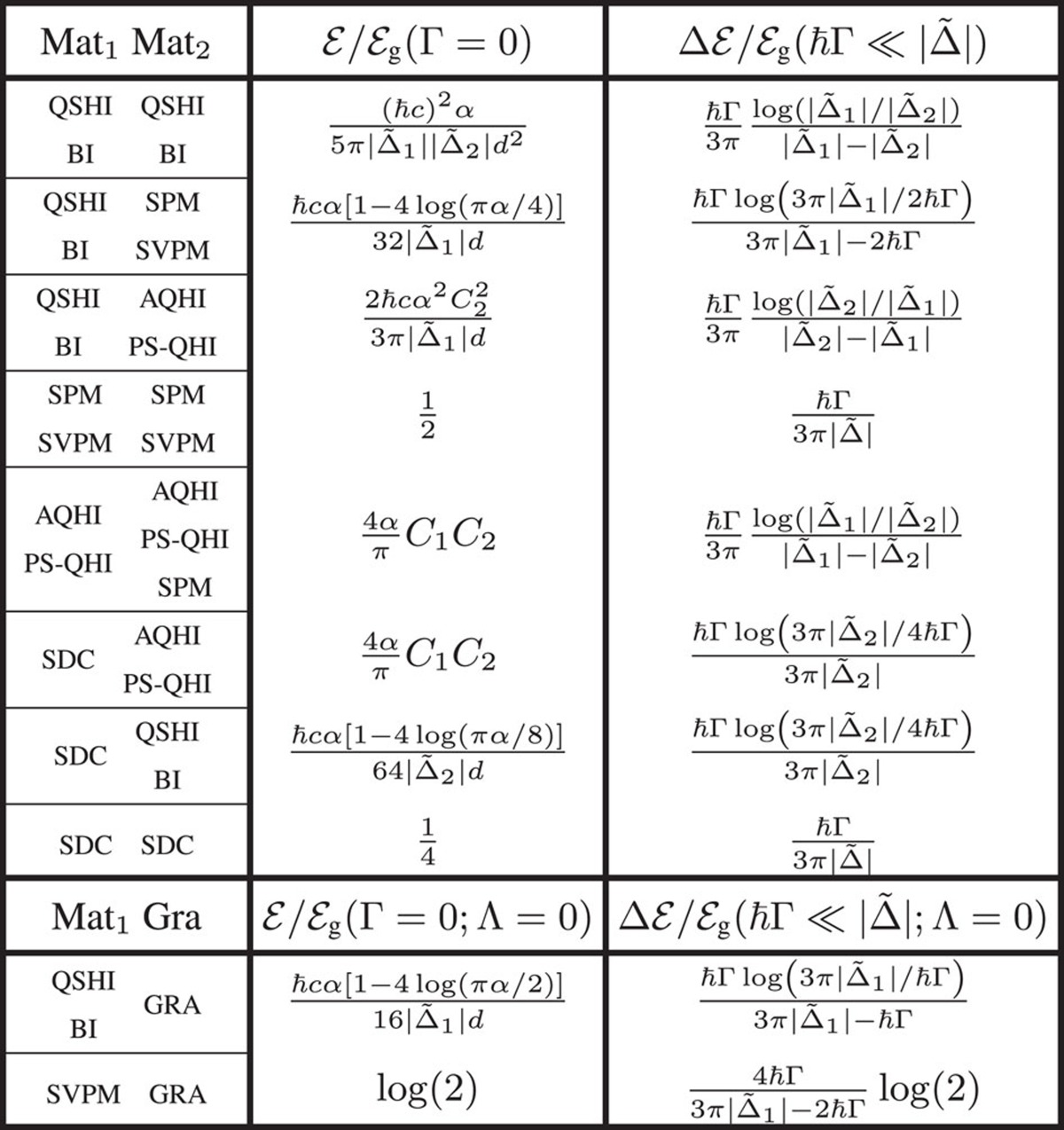
Large-distance asymptotics of the zero-temperature Casimir energy in the graphene family.

The left column denotes the phase combinations of the materials (any pair of combinations can be chosen in a given row provided they are realizable for given Λ and *E*_*z*_ values), the centre column gives the Casimir energy in the lossless case and the right column provides the correction 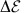 for small dissipation. When the second layer (Mat_2_) is graphene, the possible phase combinations for Λ=0 are shown in the bottom two rows, and when Λ≠0 graphene is in a AQHI phase and the possible phase combinations are given by the third and fifth rows with AQHI for Mat_2_. The inter-layer separation is large, 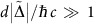, and all materials have *μ*=0 and the same Γ.
