# Supplementary material for: Casimir force phase transitions in the graphene family
Source: Nat Commun. 2017 Mar 15;8:14699. doi: 10.1038/ncomms14699 (PMC5355950; doi:10.1038/ncomms14699)
Supplement: Supplementary Information — Supplementary Notes and Supplementary Figures [file ncomms14699-s1.pdf]

Supplementary Note 1. Zero temperature optical conductivity.
The components of the optical conductivity tensor of each Dirac cone $\sigma_{ij}(i\xi, \Delta_s^\eta, \mu, T=0)$ can be written as an asymptotic series in the limit of small frequencies, as shown in Eqs. (4) in the main text. The corresponding coefficients B_{-1}^{xx}, $B_0^{xx,xy}$, and $B_1^{xx,xy}$ have intra- and inter-band contributions, i.e., $B_i^{xx} = B_i^{xx,\text{intra}} + B_i^{xx,\text{inter}}$ and $B_i^{xy} = B_i^{xy,\text{intra}} + B_i^{xy,\text{inter}}$ (here, $i = 0, \pm 1$). The intra-band coefficients are given by

$$\begin{aligned}
B_{-1}^{xx,\text{intra}} &= \frac{\mu^2 - (\Delta_s^\eta)^2}{|\mu|}\frac{\delta_{\Gamma,0}}{2\hbar}\Theta(|\mu| - |\Delta_s^\eta|)\,, \\
B_0^{xx,\text{intra}} &= \frac{\mu^2 - (\Delta_s^\eta)^2}{|\mu|}\frac{1-\delta_{\Gamma,0}}{2\hbar\Gamma}\Theta(|\mu| - |\Delta_s^\eta|)\,, \\
B_1^{xx,\text{intra}} &= \frac{\mu^2 - (\Delta_s^\eta)^2}{|\mu|}\frac{1-\delta_{\Gamma,0}}{2\hbar\Gamma^2}\Theta(|\mu| - |\Delta_s^\eta|)\,, \\
B_0^{xy,\text{intra}} &= B_1^{xy,\text{intra}} = 0,
\end{aligned} \tag{1}$$

where $\delta_{\Gamma,0}$ is the Kronecker delta and $\Theta(\Gamma)$ is the Heaviside step function. The inter-band coefficients are

$$\begin{aligned}
B_{-1}^{xx,\text{inter}} &= 0, \\
B_0^{xx,\text{inter}} &= \frac{(\Delta_s^\eta)^2}{2M\hbar\Gamma} + \left(\frac{1}{4} - \frac{(\Delta_s^\eta)^2}{\hbar^2\Gamma^2}\right)\tan^{-1}\left(\frac{\hbar\Gamma}{2M}\right), \\
B_1^{xx,\text{inter}} &= \frac{\hbar^2\Gamma^2M^2 - \hbar^2\Gamma^2(\Delta_s^\eta)^2 - 8(\Delta_s^\eta)^2M^2}{8\hbar\Gamma^2M^3 + 2\hbar^3\Gamma^4M} + \frac{2(\Delta_s^\eta)^2}{\hbar^2\Gamma^3}\tan^{-1}\left(\frac{\hbar\Gamma}{2M}\right), \\
B_0^{xy,\text{inter}} &= \frac{\eta\Delta_s^\eta}{\hbar\Gamma}\tan^{-1}\left(\frac{\hbar\Gamma}{2M}\right), \\
B_1^{xy,\text{inter}} &= \frac{\eta\Delta_s^\eta}{\hbar\Gamma^2}\left[\frac{2\hbar\Gamma M}{\hbar^2\Gamma^2 + 4M^2} - \tan^{-1}\left(\frac{\hbar\Gamma}{2M}\right)\right].
\end{aligned} \tag{2}$$

These expressions show how the individual contributions in the longitudinal and Hall conductivities of the graphene family materials depend on the Dirac mass of a particular cone, the chemical potential and scattering rate. They are especially useful for understanding how the internal properties and external factors, such as the applied electric field and circularly polarized light, determine the low-frequency electro-optical response of these materials. In Fig. 2(d) of the main text we plot these coefficients as a function of $\hbar\Gamma/|\Delta_s^\eta|$ for the case of neutral layers ($\mu = 0$). The full conductivity tensor is obtained by summing over spin and valley indices, and can be also written as in Eq. 4 of the main text with coefficients $\tilde{B}_i^{xx,xy} = \sum_{\eta,s} B_i^{xx,xy}$.

Equations 1 and 2 contain all the necessary information needed to determine the large-distance asymptotics of the zero-temperature Casimir energy between two layers of 2D staggered materials, shown in Table 1 of the main text. Using these low-frequency expansions of the conductivity tensor in the expressions for the reflection coefficients, and computing the Casimir energy as given by the Lifshitz formula to leading order in the fine structure constant, we can obtain the various entries of the table. Different phase combinations of the two layers forming the Fabry-Pérot cavity determine which B coefficients give the dominant contribution to the large-distance scaling law for the Casimir energy, as explained in the main text.

Supplementary Note 2. Finite temperature optical conductivity.
At finite temperatures the optical conductivity can be calculated via the Maldague formula [1, 2]

$$\sigma_{ij}(i\xi, \Delta_s^\eta, \mu, T) = \int_{-\infty}^{\infty} dE \frac{\sigma_{ij}(i\xi, \Delta_s^\eta, E, 0)}{4k_\text{B}T\cosh^2\left(\frac{E-\mu}{2k_\text{B}T}\right)}\,, \tag{3}$$

where $\sigma_{ij}(i\xi, \Delta_s^\eta, E, 0)$ is the zero-temperature conductivity studied in the previous Section. In Supplementary Figure 1 we show the longitudinal and Hall conductivites $\sigma_{ij}(i\xi, \mu, T) = \sum_{\eta,s}\sigma_{ij}(i\xi, \Delta_s^\eta, \mu, T)$ along imaginary frequencies for various temperatures. Panels (a-c) are the finite-temperature versions of the zero-temperature panels in Figs. 2(a-c) in the main text. For low temperatures $k_\text{B}T/\lambda_\text{SO} \ll 1$, the conductivity is essentially identical to the one at zero temperature (see Supplementary Figures 1(a,b)), except for regions in the phase-space diagram where gaps close (Supplementary Figure 1(c)). As temperature is increased, thermal effects become relevant at low frequencies, and they mainly affect the longitudinal conductivity.

In connection with the computation of the finite-temperature Casimir energy, one can see that the main effect of temperature is on the zero Matsubara frequency $\xi_{n=0}$. The conductivity at all other Matsubara frequencies $n \geq 1$ is basically unaffected by temperature. In Supplementary Figure 2 we show the variation with temperature of σ_{ij} at the zero Matsubara in all phase-space. Note that the main effect of temperature is to blur the phase transition boundaries, especially on the longitudinal conductivity.

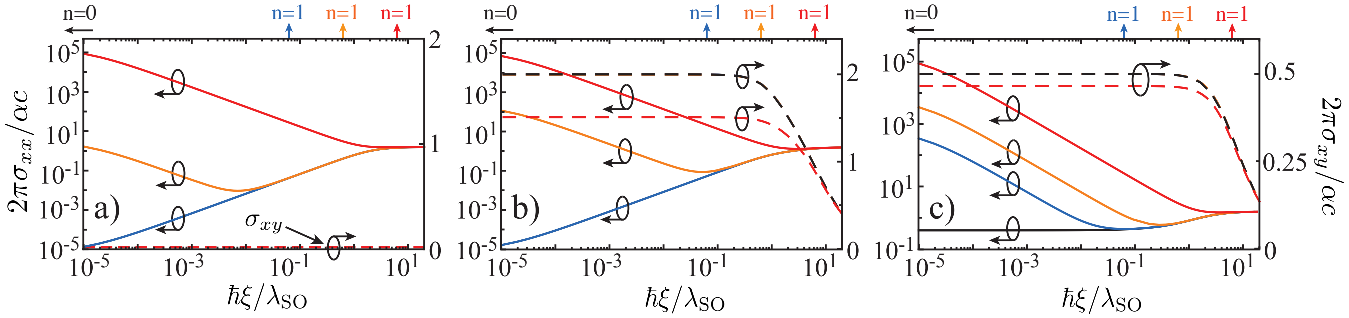

Supplementary Figure 1. **Temperature dependency of the longitudinal (solid lines) and Hall (dashed lines) conductivities at imaginary frequencies.** Temperatures $k_B T/\lambda_{SO}$ are 0 (black), 10^{-2} (blue), 10^{-1} (orange), and 1 (red). The behaviour of the conductivities along imaginary frequencies for different phases is shown: (a) $E_z = \Lambda = 0$ (QSHI phase with $C = 0$); (b) $\Lambda/\lambda_{SO} = -3/2$ and $E_z = 0$ (AQHI phase with $C = 2$); and (c) $e\ell E_z/\lambda_{SO} = -\Lambda/\lambda_{SO} = 1/2$ (SDC phase with $C = 1/2$). Due to the chosen scale, some curves are on top of each other (solid and dashed black and blue in (a) and (b); dashed black, blue, and orange in (b) and (c)). The values of the longitudinal and Hall conductivities at the $n = 0$ Matsubara are approximately equal to those corresponding to the smallest frequency shown. The position of the $n = 1$ Matsubara frequency for each temperature is shown on the top of each panel. In all cases $\mu = 0$ and $\hbar\Gamma/\lambda_{SO} = 10^{-5}$.

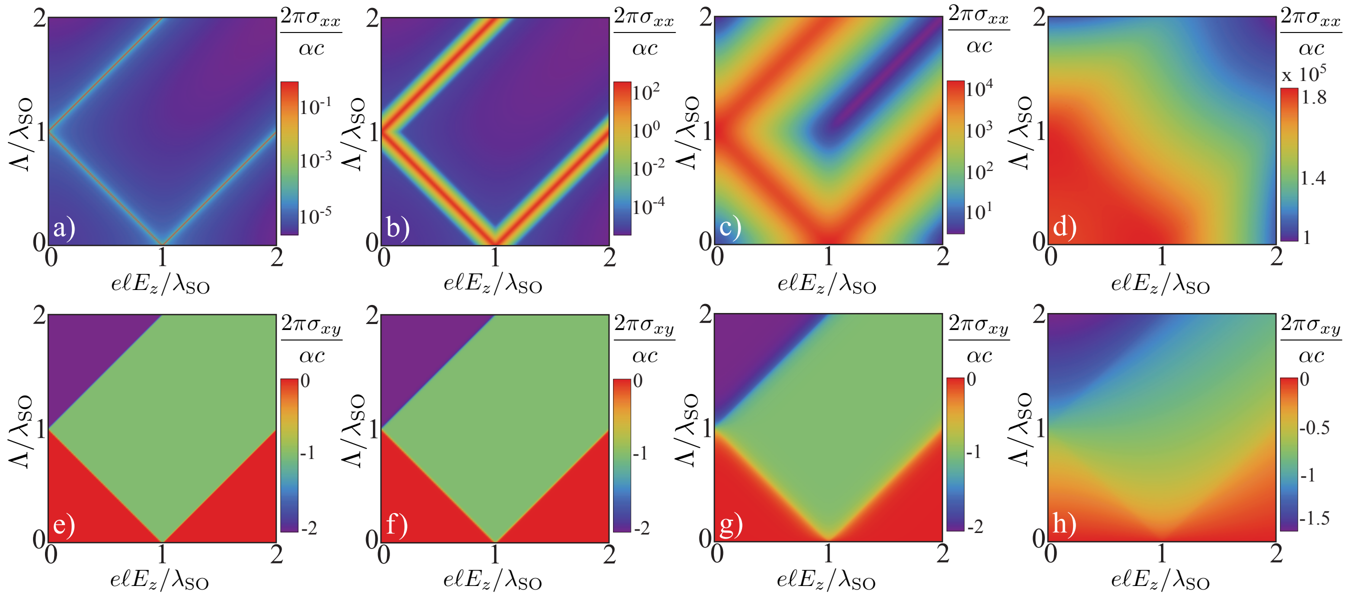

Supplementary Figure 2. **Phase diagram for the longitudinal (top panels) and Hall (bottom panels) conductivities at zero Matsubara frequency**. Temperature is $k_B T/\lambda_{SO}$: 0 (a,e), 10^{-2} (b,f), 10^{-1} (c,g), and 1 (d,h). In all cases $\mu = 0$ and $\hbar\Gamma/\lambda_{SO} = 10^{-5}$.

Supplementary Note 3. Zero-temperature Casimir energy in the graphene family.

When the chemical potential satisfies $|\mu| < |\Delta_s^\eta|$ for all Dirac cones, all intra-band B coefficients are identically zero, and the Casimir interaction is the same as that of the neutral case $\mu = 0$. When $|\mu| > |\Delta_s^\eta|$ for at least one Dirac cone, the intra-band coefficients become important. Specifically, when $\Gamma = 0$ we get $B_{-1}^{xx,\text{intra}} \neq 0$, and when $\Gamma \neq 0$ we get $B_{-1}^{xx,\text{intra}} = 0$ and $B_0^{xx,\text{intra}} > 0$. These properties are then reflected in the full coefficients $\tilde{B}_i^{xx}$ after summation over spin and valley indices. When $\tilde{B}_{-1}^{xx} > 0$, this coefficient dominates the low-frequency reflection properties of the layer, which shows a plasma-like metallic behavior. When dissipation is taken into account, the $\tilde{B}_0^{xx}$ coefficient is the first relevant one, and the layer has a Drude-like metallic behavior as long as $|\mu| \gg \hbar\Gamma$.

Supplementary Figure 3 shows the zero-temperature Casimir energy phase diagram for two identical layers of the graphene family separated by $d\lambda_{SO}/\hbar c = 1$ for $\hbar\Gamma/\lambda_{SO} = 0.0025$. Each of the panels depicts the evolution of the Casimir energy (normalized by the one for two neutral graphene sheets $\mathcal{E}_g = -\hbar c\alpha/32\pi d^3$) as the chemical potential increases. Panel 3(a) corresponds to neutral layers, $\mu = 0$, and is qualitatively the same as Supplementary Figure 3(b) of the main text. All other panels Supplementary Figure 3(b-d) satisfy $\hbar\Gamma \ll |\mu|$ and, therefore, correspond to the small dissipation limit. For $\mu/\lambda_{SO} < 1$ (3(b)) the phase diagram resembles the one of neutral layers as long as all the mass gaps are larger than the Fermi energy (for

instance, close to $E_z = \Lambda = 0$). In contrast, in regions where $|\Delta_s^\eta| < |\mu|$ the Casimir energy is largely increased due to the intra-band conductivity (orange bands in the phase diagram). For $\mu/\lambda_{\rm SO} = 1$ all points in the shown phase diagram 3(c) have at least one mass gap smaller than the chemical potential, $|\Delta_s^\eta| < |\mu|$, except along the line $\Lambda/\lambda_{\rm SO} = \ell E_z/\lambda_{\rm SO} \geq 1$ where $|\Delta_s^1| = |\mu|$ and $|\Delta_s^{-1}| > |\mu|$. Finally, for $\mu/\lambda_{\rm SO} = 2$ (panel 3(d)) the chemical potential is larger than all mass gaps in the whole phase diagram shown in the figure.

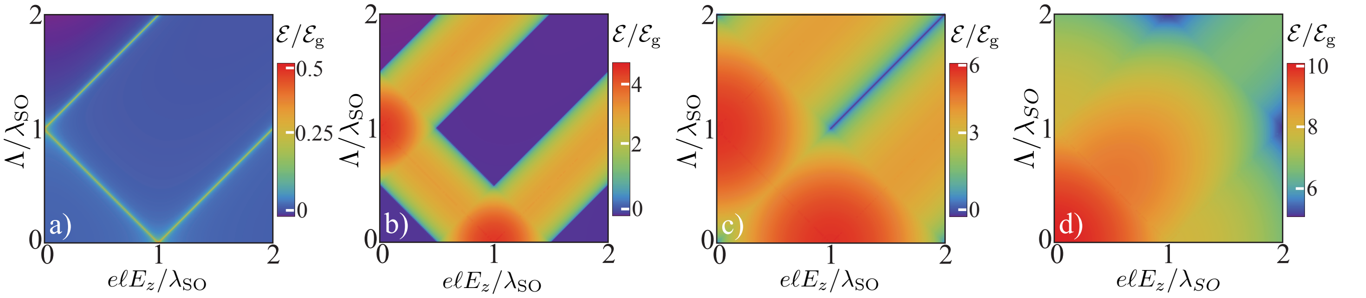

Supplementary Figure 3. **Normalized zero-temperature Casimir energy $\mathcal{E}/\mathcal{E}_{\rm g}$ phase diagram for two parallel layers for various chemical potentials.** $\mu/\lambda_{\rm SO}$ is equal to 0 (a), 0.5 (b), 1 (c), and 2 (d). Parameters are $\hbar\Gamma/\lambda_{\rm SO} = 0.0025$ and $d\lambda_{\rm SO}/\hbar c = 1$.

Supplementary Figure 4 shows how the Casimir energy phase diagram of Supplementary Figure 3(b) in the main text is modified due to finite dissipation in the materials. Losses result in the blurring of the phase boundaries and, for sufficiently large dissipation, the Casimir force is attractive for all phases.

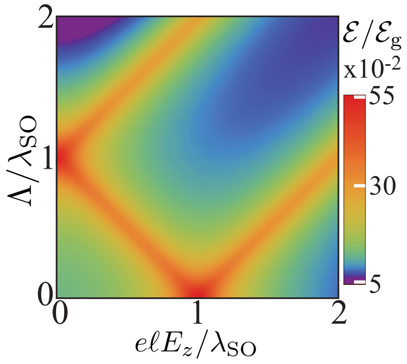

Supplementary Figure 4. **Zero-temperature Casimir energy phase diagram for two identical parallel layers of the graphene family with finite dissipation**. Parameters are $\hbar\Gamma/\lambda_{\rm SO} = 0.25$, $d = \hbar c/\lambda_{\rm SO}$, and $\mu = 0$.

We discussed in the main text the possibility of Casimir force quantization and repulsion between identical layers of the graphene family. Analogous effects take place for two dissimilar layers, as shown in Supplementary Figure 5(a) for the case of dissipationless AQHI/PS-QHI/SPM silicene and AQHI graphene phase combinations, and in Supplementary Figure 5(b) for silicene-germanene in AQHI, PS-QHI, or SPM phase combinations. Both feature a ladder-like quantized and repulsive behavior of the Casimir energy $\mathcal{E} \sim -\alpha^2 C_1 C_2 d^{-3} > 0$ with the strongest repulsion for $C_1 = -C_2 = \pm 2$. All other phase combinations result in a stronger decay with distance ($\sim d^{-4}, d^{-5}$), except for the case of silicene-graphene close to $\Lambda = 0$ and $\ell E_z/\lambda_{\rm SO}^{\rm Sil} = 1$, for which $\mathcal{E} \sim -\alpha d^{-3} < 0$. This corresponds to the attractive force between two semi-metals (SVPM silicene-graphene), and in the large-distance asymptotics results in an abrupt change of the Casimir force. Results for finite Γ are also shown in Supplementary Figures 5(c,d). Small dissipation leads to less well defined boundaries between the different phases with small peaks appearing at the steps of the ladder. Further increasing Γ makes the interaction attractive. As the interacting layers are brought closer together, the longitudinal conductivities become important ultimately resulting in Casimir attraction at shorter separations.

Supplementary Note 4. Finite-temperature Casimir energy in the graphene family.

At finite temperature, the Casimir interaction energy between two layers of the graphene family is given by

$$\mathcal{E}(T) = \frac{k_{\rm B}T}{2\pi d^2} \sum_n{}' \int_0^\infty d\tilde{k}\, \tilde{k} \log\det\left[1 - \mathbf{R}_1(c\tilde{\xi}_n/d, \tilde{k}/d) \cdot \mathbf{R}_2(c\tilde{\xi}_n/d, \tilde{k}/d)\, e^{-2\sqrt{\tilde{k}^2+\tilde{\xi}_n^2}}\right], \tag{4}$$

where the summation is over dimensionless Matsubara frequencies $\tilde{\xi}_n = 2\pi n k_{\rm B} T d/\hbar c$ ($n = 0, 1, 2, \ldots$), the prime indicates

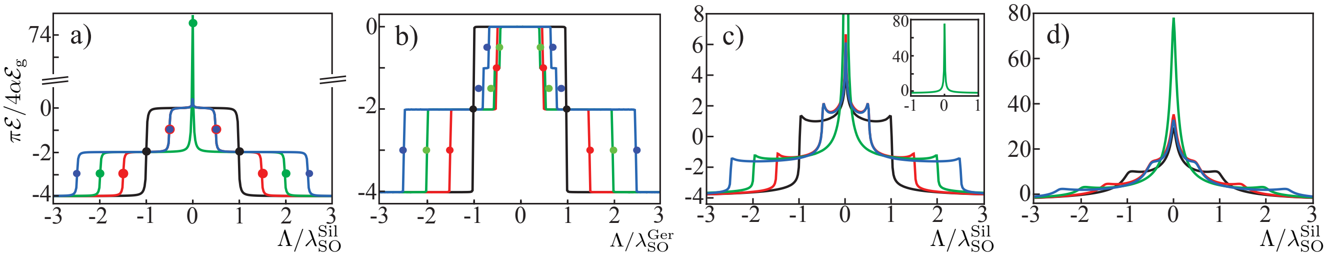

Supplementary Figure 5. **Quantized Casimir energy at zero temperature.** The normalized Casimir energy $\pi\mathcal{E}/4\alpha\mathcal{E}_{\rm g}$ is shown as a function of Λ and $\ell E_z/\lambda_{\rm SO} = \{0, 1/2, 1, 3/2\}$ (black, red, green and blue curves, respectively) for neutral and dissipationless (a) silicene-graphene ($d\lambda_{\rm SO}^{\rm Sil}/\hbar c = 10$) and (b) silicene-germanene ($d\lambda_{\rm SO}^{\rm Ger}/\hbar c = 10$). In the large-distance asymptotics (as given in Table I of the main text), the rounded plateaus become abrupt jumps and the interaction energies at phase transition boundaries are the dots in-between plateaus. The influence of dissipation for the silicene-graphene case is shown in (c) for $\hbar\Gamma/\lambda_{\rm SO}^{\rm Sil} = 0.025$ and (d) $\hbar\Gamma/\lambda_{\rm SO}^{\rm Sil} = 0.25$. The inset in (c) is a zoom-in of the energy around $\Lambda = 0$.

that the $n = 0$ term has a $1/2$ weight, and $\tilde{k} = kd$ is a dimensionless wave-vector. The reflection matrices implicitly depend on T through the temperature-dependent longitudinal and Hall conductivities.

In the limit of large distances or high temperatures (the so-called classical limit), the $n = 0$ term dominates the whole Matsubara summation, and one needs to evaluate the behavior of the reflection coefficients, and hence the conductivities, for $n = 0$. To this end, we perform a low-frequency expansion of the finite-temperature conductivities in Eq.(3), as was done for $T = 0$ in the main text, and the corresponding $\tilde{B}_i^{xx,xy}$ coefficients now depend on temperature. We separately study the cases with and without dissipation at finite temperature. When $\Gamma \neq 0$, in the limit $\xi \to 0$ we get $\sigma_{xx} \approx (\alpha c/2\pi)\tilde{B}_0^{xx}(T)$, $\sigma_{xy} \approx (\alpha c/2\pi)\tilde{B}_0^{xy}(T)$, and $R_{\rm sp}(0,\tilde{k}/d) = R_{\rm ps}(0,\tilde{k}/d) \approx 0$, $R_{\rm ss}(0,\tilde{k}/d) \approx 0$, and $R_{\rm pp}(0,\tilde{k}/d) \approx 1$. Hence, the layer behaves as a perfectly reflecting interface for p-polarized waves. Note that this is true irrespective of the point (E_z, Λ) in phase space and the value of the chemical potential. The corresponding energy is

$$\mathcal{E}_{n=0}^{\Gamma\neq 0}(T) = \frac{k_{\rm B}T}{4\pi d^2}\int_0^\infty d\tilde{k}\;\tilde{k}\log(1 - e^{-2\tilde{k}}) = -\frac{\zeta(3)}{16\pi}\;\frac{k_{\rm B}T}{d^2}, \tag{5}$$

where $\zeta(x)$ is the zeta function. When $\Gamma = 0$, in the limit $\xi \to 0$ we get $\sigma_{xx} \approx (\alpha c/2\pi)\tilde{B}_{-1}^{xx}(T)/\xi$, $\sigma_{xy} \approx (\alpha c/2\pi)\tilde{B}_0^{xy}(T)$, and the reflection coefficients $R_{\rm sp}(0,\tilde{k}/d) = R_{\rm ps}(0,\tilde{k}/d) \approx 0$, $R_{\rm pp}(0,\tilde{k}/d) \approx 1$, and $R_{\rm ss}(0,\tilde{k}/d) \approx -\kappa/(\kappa+\tilde{k})$ where $\kappa = \alpha\tilde{B}_{-1}^{xx}(T)d/c$. In contrast to the dissipative case, $R_{\rm ss}(0,\tilde{k}/d)$ is non-zero and its value is a function of the particular phase of the layer. The corresponding energy is

$$\mathcal{E}_{n=0}^{\Gamma=0}(T) = \mathcal{E}_{n=0}^{\Gamma\neq 0}(T) + \frac{k_{\rm B}T}{4\pi d^2}\int_0^\infty d\tilde{k}\;\tilde{k}\log\left(1 - \frac{\kappa^2}{(\kappa+\tilde{k})^2}e^{-2\tilde{k}}\right). \tag{6}$$

Note that $\mathcal{E}_{n=0}^{\Gamma=0}(T) \approx 2\mathcal{E}_{n=0}^{\Gamma\neq 0}(T)$ when $\kappa \gg 1$, and $\mathcal{E}_{n=0}^{\Gamma=0}(T) \approx \mathcal{E}_{n=0}^{\Gamma\neq 0}(T)$ when $\kappa \ll 1$.

[1] Giuliane, G. F., Vignale, G. *Quantum Theory of the Electron Liquid.* (Cambridge University Press, 2005).

[2] Maldague, P. F. Many-body corrections to the polarizability of the two-dimensional electron gas. *Surface Science* **73**, 296-302 (1978).
